# Poor adherence to and persistence with biologics in generalized pustular psoriasis: A claim-based study using real-world data from two large US databases

**DOI:** 10.1016/j.jdin.2023.12.008

**Published:** 2024-01-05

**Authors:** Steven R. Feldman, Rhonda L. Bohn, Ran Gao, Stephani Gray, Sabrina E. Walton, Anouk Déruaz-Luyet, Jashin J. Wu

**Affiliations:** a Department of Dermatology, Wake Forest University School of Medicine, Winston-Salem, North Carolina; b Bohn Epidemiology, Boston, Massachusetts; c Boehringer Ingelheim Pharmaceuticals Inc., Ridgefield, Connecticut; d Boehringer Ingelheim International GmbH, Ingelheim, Germany; e Department of Dermatology, University of Miami, Miller School of Medicine, Miami, Florida

**Keywords:** adherence, biologics, claims study, generalized pustular psoriasis, persistence, psoriasis

## Abstract

****Background**:**

Generalized pustular psoriasis (GPP) is a rare skin disease characterized by episodes of widespread sterile pustules.

****Methods**:**

A retrospective cohort study using data from the US IBM MarketScan Commercial and Optum Clinformatics Data Mart databases between October 1, 2015 and March 31, 2020 was performed to describe adherence and persistence to biologics in patients with GPP. Patients were aged ≥18 years with newly diagnosed GPP (International Classification of Diseases code L40.1) and had ≥1 inpatient or ≥2 outpatient claims.

**Results:**

Biologics were dispensed to 110 of 502 (22%) and 73 of 528 (14%) patients from MarketScan and Optum databases, respectively. The mean proportion of days covered (PDC) (range) was similar in both databases (MarketScan, 65% [8%-100%]; Optum, 59% [8%-99%]), and good adherence (≥80% PDC) was uncommon (MarketScan, 36%; Optum, 24%). Mean (standard deviation) persistence was similar in both databases (MarketScan, 287 [122] days; Optum, 261 [134] days). In Optum, the mean PDC was similar between age categories; good adherence was more common in patients aged 18 to 64 years (28%) versus ≥65 years (13%). Mean persistence was longer in patients aged 18 to 64 years (267 days) versus ≥65 years (242 days).

****Conclusions**:**

Overall, adherence and persistence were generally poor and varied according to the biologic class, database, and age. Improving adherence may help improve GPP treatment outcomes.

**Capsule Summary**

• Previous studies have reported sub-optimal adherence to biologics in patients with generalized pustular psoriasis.
• Here, we report poor adherence and persistence to biologics in patients with generalized pustular psoriasis; understanding the reasons for poor adherence and persistence may help improve patients’ treatment outcomes and quality of life.

## Introduction

Patients with the rare skin disease generalized pustular psoriasis (GPP), experience recurrent flares of widespread sterile pustular eruptions and erythema that can be associated with systemic inflammation.^1^ GPP has a negative impact on patients’ quality of life and can lead to life-threatening complications such as acute renal failure and congestive heart failure.^2^ Treatments for GPP have been limited historically, and medications, including biologics, have been used off-label based on their efficacy in treating plaque psoriasis.^3^ There is no globally accepted therapeutic guidance for GPP flares or long-term treatment^1^^,^^3^ and, until recently, no approved GPP-specific therapies or controlled clinical trial data. Although biologics for plaque psoriasis are approved for use in GPP in some countries, such as Japan, their approval has largely been granted based on small, open-label, single-arm studies.^4^ Consequently, the performance of these treatments is not well characterized.

Adherence is the extent to which a patient takes a medication as prescribed by their health care provider; and persistence is the time that patients remain on a medication from the first prescription to the discontinuation of treatment.^5^ Good adherence and continued persistence are key to attaining satisfactory disease control and improved quality of life, and are important for the assessment of individual treatments. Adherence to biologic treatments for psoriatic conditions can be suboptimal (38%-65%).^6^^,^^7^ In a previous study evaluating treatment patterns for patients with newly diagnosed GPP, the use of biologics was low compared with other treatment options, including oral steroids and topical agents.^8^ Here, we assess adherence to and persistence with biologic treatments in patients with GPP in the United States.

## Methods

**Fig 1:**
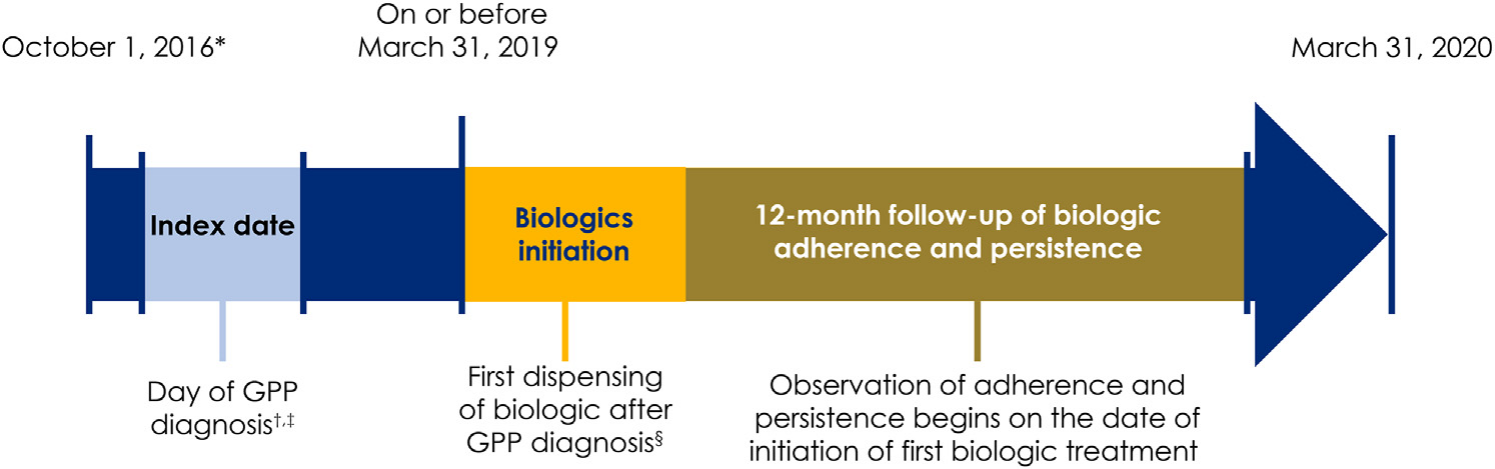
Study design of biologic adherence and persistence in GPP. ∗October 1, 2016 was chosen to permit 12 months of baseline evaluation of claims after the ICD 10th revision in 2015, which introduced a rare code for GPP; ^†^patient index dates could occur at any point during the study period, and the earliest date was October 1, 2016; ^‡^a period of 12 months prior to the index date was used to check eligibility and ensure that no claims for GPP had been made prior to this date; ^§^The date of dispensing for initial biologic treatment was also the onset of the 12-month follow-up observation of biologic adherence and perseverance. Due to the requirement for 12 months of observation, only patients whose treatment initiation occurred on or before March 31, 2019 were eligible. *GPP*, Generalized pustular psoriasis; *ICD*, International Classification of Diseases.

In this retrospective cohort study, health care claims data from 2 large databases, the IBM MarketScan Commercial Database (MarketScan) and the Optum Clinformatics Data Mart Database (Optum), were used to describe adherence to and persistence with biologic treatments in patients with newly diagnosed GPP (International Classification of Diseases [ICD] 10^th^ Revision code L40.1). Data were collected between October 1, 2015 and March 31, 2020, and the patient-selection period was from October 1, 2016 to March 31, 2020 (Fig 1).

Included patients were aged ≥18 years and had ≥1 inpatient or ≥2 outpatient claims that were 30 to 180 days apart, with a GPP diagnosis (ICD code L40.1). For the adherence and persistence analyses, only patients with ≥12 months' continuous enrollment in the database after the initial dispensing of biologics were included. Biologics included tumor necrosis factor-alfa (TNF-α) inhibitors (adalimumab, etanercept, certolizumab pegol, infliximab, and golimumab), interleukin (IL) inhibitors (secukinumab, ustekinumab, brodalumab, ixekizumab, guselkumab, tildrakizumab, and risankizumab), and T-cell inhibitors (abatacept).

Adherence to biologics was measured as the proportion of days covered (PDC), which was calculated by the number of days in the follow-up period that a patient had a specific biologic treatment available (ie, number of days with a filled prescription) divided by the total days in the follow-up period, measured over a 12-month period (Fig 1).^9^ Good adherence was defined as ≥80% PDC.^9^ Persistence was defined as the number of days from biologic initiation to treatment discontinuation (date of last fill plus the days supplied) or the end of follow-up. Considering how infrequently biologic treatments are prescribed, a gap of ≤84 days between subsequent dispensing of biologics was permitted and was considered as continuous use. Adherence and persistence were stratified by age in the Optum database into 2 categories: 18- to 64-year-olds and >65-year-olds. There are limited data for patients aged ≥65 years in MarketScan due to its structure, preventing such analyses with these data. All analyses were purely descriptive, with no formal statistical comparisons made.

## Results

In total, 502 (MarketScan) and 528 (Optum) patients with GPP were identified, of whom 110 (22%) (MarketScan) and 73 (14%) (Optum) were treated with biologics and met all inclusion criteria (Table I). Most patients were treated with TNF-α inhibitors (MarketScan, *N* = 75 [68%]; Optum, *N* = 46 [63%]); under half received IL inhibitors (MarketScan, *N* = 49 [45%]; Optum, *N* = 32 [44%]); and a few received T-cell inhibitors (MarketScan, *N* = 2 [2%]; Optum, *N* = 4 [5%]). Mean overall PDC for biologics was similar between databases (MarketScan, 65% [range, 8%-98%]; Optum, 59% [range, 8%-99%]). A minority of patients showed good adherence in the MarketScan (36%) and Optum (25%) databases (Table I). When assessed according to the class of biologics, mean PDC and good adherence varied (Table I). Mean (standard deviation [SD]) biologic persistence was comparable between databases (MarketScan, 287 [122] days; Optum, 261 [134] days). However, there was some variance between drug classes: TNF-α inhibitors (MarketScan, 255 [127] days; Optum, 214 [138] days), IL inhibitors (MarketScan, 288 [122] days; Optum, 265 [133] days), and T-cell inhibitors (MarketScan, 211 [219] days; Optum, 299 [114] days) (Table I).

**Table I:** Biologic adherence and persistence for MarketScan and Optum databases

Assessment	MarketScan (*N* = 110)			Optum (*N* = 73)		
	*n*	Mean (SD)	Adherence ≥80%, *n* (%)	*n*	Mean (SD)	Adherence ≥80%, *n* (%)
PDC, %						
Any biologic treatment	110	65 (25)	40 (36.4)	73	59 (27)	18 (24.7)
TNF-α inhibitors	75	57 (27)	20 (26.7)	46	49 (26)	7 (15.2)
IL inhibitors	49	67 (25)	19 (38.8)	32	62 (27)	10 (31.3)
T-cell inhibitors	2	41 (36)	0 (0.0)	4	62 (25)	1 (25.0)
Persistence with initial biologic treatment, days from start to discontinuation						
Any biologic treatment	110	287 (122)		73	261 (134)	
TNF-α inhibitors	75	255 (127)		46	214 (138)	
IL inhibitors	49	288 (122)		32	265 (133)	
T-cell inhibitors	2	211 (219)		4	299 (114)	

Patients may have received more than 1 biologic treatment during the 12-month follow-up period.

*IL*, Interleukin; *PDC*, proportion of days covered; *SD*, standard deviation; *TNF*, tumor necrosis factor.

We conducted a sub-analysis to assess biologic adherence and persistence between age groups in the Optum database. There were 268 patients aged 18 to 64 years and 260 patients aged ≥65 years, of whom 57 (21%) and 16 (6%) were treated with biologics, respectively. Mean PDC was similar for both cohorts (18 to 64 years, 60% [range, 8%-99%]; ≥65 years, 56% [range, 8%-96%]; Table II). Good adherence was higher in the 18- to 64-year-old cohort at 28% versus 13% in the ≥65-year-old cohort. Mean PDC and good adherence across drug classes varied between age groups, although patient numbers were low. Overall persistence (mean [SD]) was longer in the 18- to 64-year-old cohort compared with the ≥65-year-old cohort (18 to 64 years, 267 [130] days; ≥65 years, 242 [151] days). However, persistence varied by biologic drug class and was shorter in patients aged 18 to 64 years for T-cell inhibitors (227 [154] vs 366 [non-calculable] days) and slightly shorter for TNF-α inhibitors (206 [135] vs 232 [149] days) compared with the older patient category. Notably, only 1 patient in the ≥65-year-old cohort was treated with T-cell inhibitors. Patients in the 18- to 64-year-old cohort (278 [SD 127] days) persisted with IL inhibitors for longer compared with the ≥65-year-old cohort (180 [SD 165] days).

**Table II:** Biologic adherence and persistence for Optum age groups (18 to 64 and ≥65 years)

Assessment	18 to 64 years (*N* = 57)			≥65 years (*N* = 16)		
	*n*	Mean (SD)	Adherence ≥80%, *n* (%)	*n*	Mean (SD)	Adherence ≥80%, *n* (%)
PDC, %						
Any biologic treatment	57	60 (28)	16 (28.1)	16	56 (23)	2 (12.5)
TNF-α inhibitors	33	48 (27)	6 (18.2)	13	52 (25)	1 (7.7)
IL inhibitors	28	63 (27)	9 (32.1)	4	51 (28)	1 (25.0)
T-cell inhibitors	3	55 (26)	0 (0.0)	1	80 (N/A)	1 (100.0)
Persistence with initial biologic treatment, days from start to discontinuation						
Any biologic treatment	57	267 (130)		16	242 (151)	
TNF-α inhibitors	33	206 (135)		13	232 (151)	
IL inhibitors	28	278 (127)		4	180 (165)	
T-cell inhibitors	3	227 (154)		1	366 (N/A)	

Patients may have received more than 1 biologic treatment during the 12-month follow-up period.

*IL*, Interleukin; *N/A*, not applicable; *PDC*, proportion of days covered; *SD*, standard deviation; *TNF*, tumor necrosis factor.

## Discussion

This retrospective cohort study used US claims data to assess biologic adherence and persistence in patients with GPP. Low adherence and persistence were largely consistent between the MarketScan and Optum databases, with some variation between biologic classes and age groups. A minority of patients in MarketScan and Optum achieved good adherence, and only 1 treatment class (T-cell inhibitors) had an average of 366 days' persistence, although this was only achieved by 1 older patient, which precludes broader generalizations.

Our current analysis suggests that patients with GPP do not use biologics over the long term, as adherence to biologics was low and only 1 patient persisted with treatment beyond 1 year. A similar study on the adherence and persistence of biologics in patients with psoriasis showed that good adherence ranged between 18% and 44% and persistence ranged from 181 to 358 days.^5^ A German study of patients with GPP measured time to biologic discontinuation as 13 to 36 months.^10^ However, as this study assessed medical records, rather than claims data, and came from a different health care system, results may not be directly comparable.

There are several possible reasons for the low biologic adherence and persistence observed in our study. Patients with GPP frequently switch medications over time, perhaps due to a lack of efficacy.^8^ Treatment ineffectiveness is a common reason for the cessation of biologics in GPP,^10^ as are biologic-associated adverse events.^11^ Additionally, GPP is a predictor for biologic treatment discontinuation.^11^ Because GPP can be a relapsing disease with recurrent flares, treatment discontinuation may occur after flare symptomatic improvement. The presence of comorbidities may also have influenced the types of medications received and continuation of these medications, particularly in older patients for whom factors such as an increased risk of infection with immunosuppressive biologic therapies as well as an increased risk of complications from systemic treatments may impact treatment options.^12^ We have previously shown the burden of comorbidities in patients with GPP in these databases.^8^ A major limitation of using claims databases to assess biologic adherence and persistence is that claims databases do not collect data on the reasons for treatment discontinuation; thus, the relative contribution of each of the above factors cannot be quantified at a granular level. As a result, it is unknown which factors are most likely to negatively affect biologic adherence and persistence, and what the actual adherence and persistence is. Reasons for hospitalization, which could be an indicator of increased disease severity, were also not disclosed in this study, so no conclusions could be made about how the incidence of GPP flares may influence biologic adherence and persistence.

In addition to the important limitations described above, the primary limitation of this study is that the omission of treatment data for patients with <12 months of follow-up after the initiation of a biologic treatment may have introduced biases and restricted the number of patients who could be included in these analyses. However, as GPP requires long-term management, the inclusion of patients with <12 months of follow-up after initiation of a biologic treatment could have skewed mean adherence, as patients may not have discontinued treatment but continued with dispensing through another provider. Another study limitation was the potential for inaccurate, incomplete, or missing data inherent to administrative databases. Despite the limitations associated with using claims data, the Optum and MarketScan databases are both large enough to be considered representative of the population of commercially insured patients in the United States. An additional limitation was the absence of documented reasons for dispensing, meaning it was not possible to establish whether the medications were dispensed for the treatment of GPP. Specifically, many treatments approved to treat plaque psoriasis are often used to treat GPP, despite GPP now being recognized as a disease distinct from plaque psoriasis.^13^ Therefore, as new GPP-specific biologic treatments are approved, discontinuation rates may lessen compared with biologics that were designed primarily to target plaque psoriasis. Recently, the anti-IL-36 receptor antibody spesolimab gained approval in the United States,^14^ Europe,^15^ Japan,^16^ and China^17^ for the treatment of GPP flares, based on a placebo-controlled, randomized Phase II trial (NCT03782792). Another placebo-controlled, randomized Phase II trial (NCT04399837) demonstrated that high-dose spesolimab is effective at preventing GPP flares versus placebo.^18^ These data may result in the approval of spesolimab as a GPP-specific maintenance therapy.

## Conclusion

In conclusion, adherence to and persistence with biologics were both poor in patients with GPP in these databases. There are several important influencing factors, including treatment efficacy and treatment switching, presence of comorbidities, and age, among others, which may contribute to this. Further research is needed in other GPP populations to understand if the results of the present study are generalizable, and to understand the granular reasons for these findings. This will also be important for improving future treatment outcomes for patients with GPP. It will be interesting to see whether the introduction of new GPP-specific treatments will improve adherence and persistence in the future.

## Conflicts of interest

Steven R. Feldman declares receiving research, speaking, and/or consulting support from AbbVie, Accordant, Advance Medical, Almirall, Alvotech, Amgen, Arcutis, Arena Pharmaceuticals, Argenx, Biocon, Boehringer Ingelheim, Bristol Myers Squibb, Caremark, Celgene, Dermavant, Eli Lilly, Galderma, GlaxoSmithKline/Stiefel, Helsinn, Informa, Janssen, LEO Pharma, Menlo, Merck, Mylan, National Biological Corporation, National Psoriasis Foundation, Novan, Novartis, Pfizer, Qurient, Regeneron, Samsung, Sanofi, Sun Pharmaceutical Industries, Suncare Research Laboratories, UCB, UpToDate, Valeant, and vTv Therapeutics. He is also the founder and majority owner of www.DrScore.com and has stock in Sensal. Rhonda L. Bohn is the founder of Bohn Epidemiology, LLC and has served as a consultant to Boehringer Ingelheim. Ran Gao is a former employee of Boehringer Ingelheim and is now an employee of Gilead Sciences. Stephani Gray declares being a consultant to Boehringer Ingelheim and Bohn Epidemiology, LLC. Sabrina E. Walton declares being an employee of Boehringer Ingelheim. Anouk Déruaz-Luyet declares being an employee of Boehringer Ingelheim. Jashin J. Wu declares being an investigator, consultant, or speaker for AbbVie, Almirall, Amgen, Arcutis, Aristea Therapeutics, Bausch Health, Boehringer Ingelheim, Bristol Myers Squibb, Dermavant, DermTech, Dr. Reddy’s Laboratories, Eli Lilly, EPI Health, Galderma, Janssen, LEO Pharma, Mindera, Novartis, Pfizer, Regeneron, Samsung Bioepis, Sanofi Genzyme, Solius, Sun Pharmaceutical Industries, UCB, and Zerigo Health.
